# Reduction of the Nitro Group to Amine by Hydroiodic Acid to Synthesize *o*-Aminophenol Derivatives as Putative Degradative Markers of Neuromelanin

**DOI:** 10.3390/molecules19068039

**Published:** 2014-06-16

**Authors:** Kazumasa Wakamatsu, Hitomi Tanaka, Keisuke Tabuchi, Makoto Ojika, Fabio A. Zucca, Luigi Zecca, Shosuke Ito

**Affiliations:** 1 Department of Chemistry, Fujita Health University School of Health Sciences, Toyoake, Aichi 470-1192, Japan; E-Mails: hmatsuoka@u-gifu-ms.ac.jp (H.T.); 82013103@fujita-hu.ac.jp (K.T.); sito@fujita-hu.ac.jp (S.I.); 2 Department of Applied Molecular Biosciences, Graduate School of Bioagricultural Sciences, Nagoya University, Furo-cho, Chikusa-ku, Nagoya 464-8601, Japan; E-Mail: ojika@agr.nagoya-u.ac.jp; 3 Institute of Biomedical Technologies, National Research Council of Italy, Via Cervi, 93, Segrate, Milano 20090, Italy; E-Mails: fabio.zucca@itb.cnr.it (F.A.Z.); luigi.zecca@itb.cnr.it (L.Z.)

**Keywords:** neuromelanin, aminohydroxyphenylethylamine, aminohydroxyphenylacetic acid, aminohydroxyethylbenzene, Parkinson’s disease, HI reduction

## Abstract

Neuromelanin (NM) is produced in dopaminergic neurons of the substantia nigra (SN) and in noradrenergic neurons of the locus coeruleus (LC). The synthesis of NM in those neurons is a component of brain aging and there is the evidence that this pigment can be involved in the pathogenesis of neurodegenerative diseases such as Parkinson’s disease. NM is believed to derive from the oxidative polymerization of dopamine (DA) or norepinephrine (NE) with the participation of cysteine, dolichols and proteins. However, there are still unknown aspects in the chemical structure of NM from SN (SN-NM) and LC (LC-NM). In this study, we designed a new method to synthesize *o*-aminophenol compounds as putative degradation products of catecholamines and their metabolites which may be incorporated into NM. Those compounds are aminohydroxyphenylethylamine (AHPEA) isomers, aminohydroxyphenylacetic acid (AHPAA) isomers and aminohydroxyethylbenzene (AHEB) isomers, which are expected to arise from DA or NE, 3,4-dihydroxyphenylacetic acid (DOPAC) or 3,4-dihydroxyphenylmandelic acid (DOMA) and 3,4-dihydroxyphenylethanol (DOPE) or 3,4-dihydroxyphenylethyleneglycol (DOPEG), respectively. These *o*-aminophenol compounds were synthesized by the nitration of phenol derivatives followed by reduction with hydroiodic acid (HI), and they could be identified by HPLC in HI hydrolysates of SN-NM and LC-NM. This degradative approach by HI hydrolysis allows the identification of catecholic precursors unique to SN-NM and LC-NM, which are present in catecholaminergic neurons.

## 1. Introduction

There are two chemically distinct types of peripheral melanins, black to brown pigments called eumelanin, and yellow to reddish-brown pigments called pheomelanin [1,2,3]. Eumelanin is derived from the oxidative polymerization of 5,6-dihydroxyindole (DHI) and 5,6-dihydroxyindole-2-carboxylic acid (DHICA), while pheomelanin consists of benzothiazine and benzothiazole units arising from cysteinyl-3,4-dihydroxyphenylalanine (Cys-DOPA) isomers. On the other hand, neuromelanin (NM) consists of black to brown pigments mainly found in neurons of the substantia nigra (SN) and locus coeruleus (LC) in the central nervous system of human and other mammalian species [4]. Previous studies from our group and other groups have shown that NM consists of complex polymers derived from eumelanic DHI and pheomelanic benzothiazine units along with additional aliphatic and proteic components [5,6,7,8]. NM accumulates during normal aging in neurons of different brain areas but, interestingly, SN and LC that are the main target regions of Parkinson’s disease (PD) are those with the highest pigment concentrations [9,10]. NM is formed by the oxidation of catecholamines, dopamine (DA) and norepinephrine (NE), in the presence of cysteine (Cys). The synthesis of NMs in the various regions of the human brain is an important protective process because the melanic components are generated through the removal of reactive or toxic *o*-quinones that would otherwise cause neurotoxicity. Another important aspect of the protective role of NM is the ability to bind toxic metals forming stable complexes thereby blocking their toxicity [10]. On the other hand, due to its biochemical properties, NM has also been discussed as a critical factor underlying neuronal vulnerability in PD [5,9,10,11]. In PD the NM released by degenerating neurons can cause microglia activation and neurodegeneration. In cell culture and *in vivo* studies it was shown that human NM induces microglia activation and neuronal death [11,12,13,14]. In SN and LC, catecholamine neurons containing NM highly express major histocompatibility complex-I which triggers neuronal death mediated by cytotoxic T cells [15].

To study the structure and function of peripheral (cutaneous) melanin, we have developed several chemical methods to quantify eumelanin and pheomelanin [16,17,18,19]. Alkaline H_2_O_2_ oxidation affords pyrrole-2,3,5-tricarboxylic acid (PTCA) as a eumelanin marker and thiazole-2,4,5-tricarboxylic acid (TTCA) as a pheomelanin marker. Pheomelanin can also be analyzed as 4-amino-3-hydroxyphenylalanine (4-AHP) after hydroiodic acid (HI) hydrolysis. To study the structure and function of NM, we used HPLC to analyze 4-amino-3-hydroxyphenylethylamine (4-AHPEA) and 3-amino-4-hydroxyphenylethylamine (3-AHPEA) after HI hydrolysis, in addition to pyrrole-2,3-dicarboxylic acid (PDCA), TTCA and thiazole-4,5-dicarboxylic acid (TDCA) after H_2_O_2_ oxidation. Several markers analyzed by HPLC are currently used for melanin analysis, but only two, PTCA and 3-amino-4-hydroxyphenylalanine (3-AHP), are commercially available. Thus, most of those HPLC markers must be prepared by simple chemical processes [20]. 4-AHP, a major degradation product of pheomelanin upon HI hydrolysis, can be prepared by HI hydrolysis of 5-*S*-cysteinyldopa-melanin [20,21]. However, we found that this amino acid can be more readily obtained in a 100 mg scale by the nitration of commercially available *m*-tyrosine followed by HI reduction of the resulting 3-hydroxy-4-nitrophenylalanine along with other possible isomers [20]. Authentic samples of 4-AHPEA and 3-AHPEA used in previous studies were prepared by reductive HI hydrolysis of 5-*S*-cysteinyldopamine-melanin (5-*S*-CysDA-melanin) and 2-*S*-cysteinyldopamine-melanin (2-*S*-CysDA-melanin) according to established methods [6,7,22,23]. However, this method requires the preparation of 5-*S*-CysDA and 2-*S*-CysDA in advance followed by their oxidation to melanin, which is time-consuming and requires the skillful separation of 5-*S*-CysDA and 2-*S*-CysDA. Thus, an alternative chemical method was needed to prepare these *o*-aminophenol derivatives in more convenient and generally applicable manner. In this study, we developed a convenient reduction of the nitro group to amine with HI to synthesize *o*-aminophenol derivatives as putative degradative markers of human NM.

## 2. Results and Discussion

### 2.1. Chemistry

**Scheme 1:**
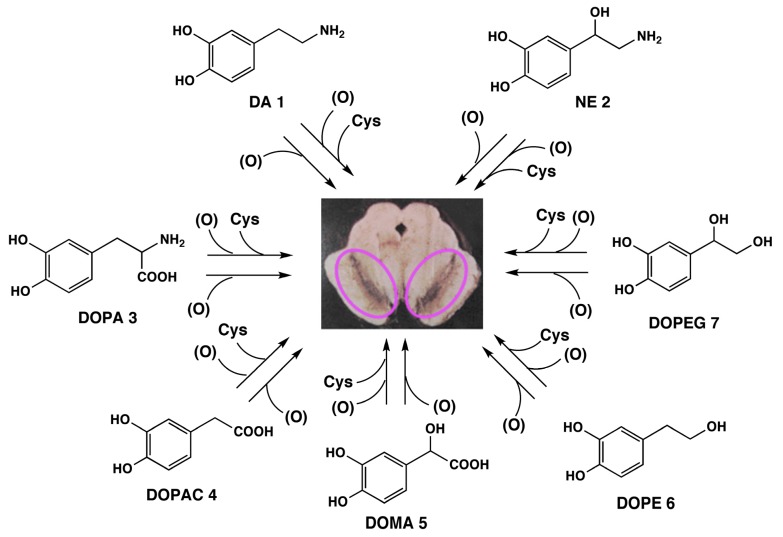
Possible participation in NM biosynthesis of various catecholic metabolites known to be present in various brain regions of the human brain. Purple circles depict NM accumulation in neurons of the SN. (O) represents oxidants.

**Scheme 2:**
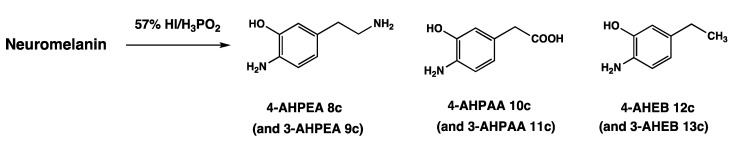
Possible degradation products afforded by the reductive HI hydrolysis of human NM.

The biosynthesis of NM, oxidative polymerization of catecholamines, suggests the possibility that various catecholic metabolites would also participate in NM genesis (Scheme 1). In addition to **1** and **2**, 3,4-dihydroxyphenylalanine (DOPA, **3**), 3,4-dihydroxyphenylacetic acid (DOPAC, **4**), 3,4-dihydroxymandelic acid (DOMA, **5**), 3,4-dihydroxyphenylethanol (DOPE, **6**) and 3,4-dihydroxy-phenylethyleneglycol (DOPEG, **7**) can be incorporated into NM. These compounds are metabolites of **1** and **2** formed by oxidative deamination by monoamine oxidase followed by oxidation/reduction. We also reported that melanin pigments derived from Cys-DOPA can be identified in NMs isolated from other important regions of human brain, such as putamen, premotor cortex, and cerebellum [10]. We have already established methods to determine eumelanin and pheomelanin in peripheral (cutaneous) melanin that can be used for the characterization of NM [7,8,16,17,18,19,20]. These methods are based on melanin degradation by alkaline H_2_O_2_ oxidation and reductive HI hydrolysis. Upon HI hydrolysis, NM afforded 4-AHPEA **8c** and its isomer 3-AHPEA **9c** along with benzothiazole derivatives. These *o*-aminophenol compounds are derived from Cys-DA and Cys-NE melanins. HI hydrolysis of NMs, derived from the above catechol derivatives, **1**–**7**, is expected to afford the corresponding *o*-aminophenol derivatives, AHPEAs (**8c** and **9c**), aminohydroxyphenylacetic acid (AHPAAs, **10c** and **11c**), and aminohydroxyethylbenzene (AHEBs, **12c** and **13c**) (Scheme 2).

**Scheme 3:**
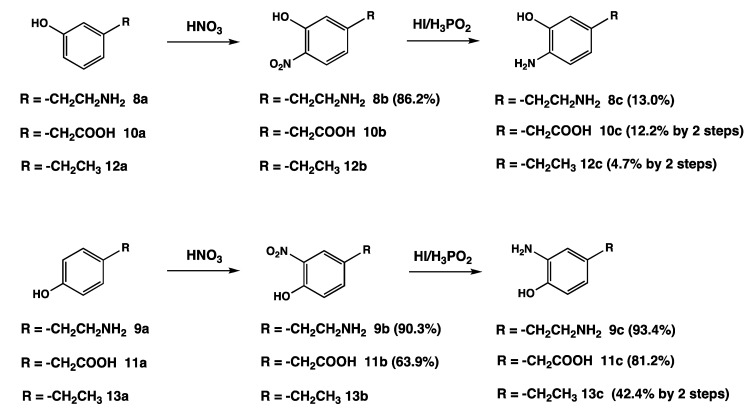
Synthetic scheme of the nitration of *m*- and *p*-substituted phenol derivatives followed by reduction of the nitro group to amine with reductive HI hydrolysis. Parenthesis is a yield.

Thus, we planned a synthetic scheme involving the *o*-nitration of the corresponding phenols with 60% HNO_3_ followed by reduction with 57% HI. The desired *o*-aminophenols **c** were separated on a cation exchange resin and purified by recrystallization of the HCl salts (Scheme 3).

The synthesis of the *o*-aminophenol derivatives **c** comprises a series of two known reactions (Scheme 3). The first step (aromatic nitration) and the second step (reduction of the nitro group to amine) are common to all products **c**. In the first step, the nitration of phenols **a** was carried out by 60% HNO_3_ at room temperature for 10 min—overnight followed by separation of the nitrated phenols with a cation exchange resin or extraction with ethyl acetate. *o-* and *p*-Oriented nitrophenol isomers were subjected to reduction of the nitro group without further purification. The nitration of *p*-substituted phenols afforded only *o*-nitrophenol derivatives **b**, so the yields were nearly quantitative. On the other hand, the nitration of *m*-substituted phenols afforded isomeric *o*- and *p*-oriented nitrophenols and dinitrophenol derivatives. Thus, the yields of the target compounds were much lower than those from *p*-substituted phenol derivatives.

In the literature, several methods for the reduction of nitro groups to amines have been reported. Aromatic amines are known to be produced by catalytic hydrogenation under continuous high pressure [24,25,26,27]. Nitro compounds can also be reduced to amines in good yields with iron and hydrochloric acid in the Béchamp reaction [25], which is the oldest commercial process for preparing amines, and is still used in the dyestuff industry. The reduction of *o*-nitrophenol **b** to *o*-aminophenol **c** must be done under a reductive environment because aminophenol is susceptible to oxidation. Thus, we have developed the HI/H_3_PO_2_ method for reduction of the nitro group. HI has a high acidity and potential reducing ability. Compared with other reduction methods in the literature, this method is easy because the work-up after the reaction requires only the removal of HI under reduced pressure without further filtration of the catalyst or extraction of the solution. After the evaporation of HI, the reaction mixture was chromatographed on an ion-exchange resin. The target compound as the HCl salt was further recrystallized from ethanol plus ether. 3-Amino-4-hydroxyphenyl derivatives **9c**, **11c** and **13c** were obtained in moderate to high overall yields (84%, 52% and 42%, respectively). On the other hand, 4-amino-3-hydroxyphenyl derivatives **8c**, **10c** and **12c** were obtained in relatively low overall yields (11%, 12% and 4.7%, respectively), because of the production of other possible isomers and diamino derivatives. The poor yield of **12c** may be due to the low purity (80%) of the starting material, 3-ethylphenol (**12a**) which contains the isomers 4-ethylphenol (**13a**) and 2-ethylphenol making the separation of the desired product difficult. As the isolation of **10b**, **12b**, and **13b** were difficult, the reaction products were subjected to the following reaction without the further purification.

### 2.2. Chemical Analyses of NM Isolated from the SN and LC of Human Brain

The reductive HI hydrolysis of NM isolated from the SN and LC of human midbrains was performed, and the degradation products were identified by HPLC using newly synthesized *o*-aminophenol compounds **c** as standards (Table 1). It should be emphasized that LC-NM has never been studied before by chemical degradative methods, which have been mainly employed on NM isolated from other areas of human brain [6,7,10]. We have previously reported that the chemical degradation of SN with HI affords **8c** and **9c** in high yields, and **14c** and **15c** in low yield [6,7,10]. AHP isomers **14c** and **15c** are derived from Cys-DOPA melanin [18]. In this study, we could detect AHEBs for the first time as a mixture of **12c** and **13c**, and AHPAA isomers **10c** and **11c** in SN-NM. In LC-NM, we could also now detect **8c**, **9c**, **10c** and **11c** together with AHEBs **12c** and **13c**. These results suggest that SN-NM and LC-NM are derived not only from DA **1** and NE **2**, but also from DOPA **3**, DOPAC **4**, DOMA **5**, DOPE **6** and DOPEG **7**, because AHPEA isomers **8c**, **9c**, AHPAA isomers **10c**, **11c**, AHEB isomers **12c**, **13c** and AHP isomers **14c**, **15c** are expected to arise from DA **1** or NE **2**, DOPAC **4** or DOMA **5**, DOPE **6** or DOPEG **7** and DOPA **3**, respectively. We could also detect DA **1**, DOPA **3**, and DOPAC **4** after HI hydrolysis. This result indicated that the protein-bound derivatives of DA **1**, DOPA **3**, and DOPAC **4** were incorporated into SN-NM and LC-NM [10].

**Table 1:** Chemical characterization with reductive HI hydrolysis of human NM isolated from SN and LC tissues (values in ng/mg).

Sample	DA (1)	DOPA (3)	DOPAC (4)	4-AHPEA (8c)	3-AHPEA (9c)	4-AHPAA (10c)	3-AHPAA (11c)	AHEBs (12c, 13c)	4-AHP (14c)	3-AHP (15c)
SN-NM	1,224	722	161	428	588	735	571	32	62	66
LC-NM	955	624	368	806	695	596	310	75	52	114

Values were obtained in a single determination.

## 3. Experimental Section

### 3.1. General Information

^1^H-NMR spectra (400 MHz for ^1^H-NMR and 100 MHz for ^13^C-NMR) were recorded on a Bruker AVANCE 400 spectrometer and are reported as the chemical shifts δ (ppm) downfield from sodium 2,2-dimethyl-2-silapentane-5-sulfonate (DSS) or *t*-butyl alcohol used as an internal reference. Coupling constants (*J*) are expressed in Hz and signals are expressed as s (singlet), d (doublet) or t (triplet). Elemental analyses were carried out by the Organic Elemental Analysis Research Center (Kyoto University, Japan). For ion-exchange column chromatography, a Dowex 50W-X2 (200–400 mesh) column was used. UV spectrometry was monitored using a JASCO V-630 UV-VIS spectrometer (JASCO Co., Tokyo, Japan). HPLC systems consisted of a JASCO 880-PU pump, a JASCO C_18_ column (JASCO Catecholpak; 4.6 mm × 150 mm; 7 µm particle size) and an EICOM ECD-300 electrochemical detector (EICOM, Kyoto, Japan), and a Shiseido C_18_ column (Shiseido Capcell Pak MG; 4.6 mm × 250 mm; 5 µm particle size) and a JASCO UV detector (JASCO Co.). DA **1**, NE **2**, DOMA **5**, DOPEG **7**, 3-amino-4-hydroxyphenylalanine (3-aminotyrosine, **15c**), 57% HI and mushroom tyrosinase (4,276 U/mg) were purchased from Sigma Aldrich Japan (Tokyo, Japan), 3-ethylphenol **12a** (80% purity), 4-ethylphenol **13a** and DOPE **6** were purchased from Tokyo Chemical Industry Co. (Tokyo, Japan), and 3-hydroxyphenylethylamine **8a**, 4-hydroxyphenylethylamine **9a**, 3-hydroxyphenylacetic acid **10a**, 4-hydroxyphenylacetic acid **11a** and Dowex 50W-X2 were purchased from Wako Pure Chemical Industries, Ltd. (Osaka, Japan). All other chemicals were of the highest purity available.

Reductive HI hydrolysis was performed as described previously [6,7,18,20]. Briefly, a mixture of 100 μL suspension of a sample (containing 0.1 mg melanin), 30 μL 30% H_3_PO_2_ and 500 μL 57% HI was heated in a screw-capped tube at 130 °C for 20 h, after which the mixture was cooled. An aliquot (100 μL) of each hydrolysate was transferred to a test tube and evaporated to dryness using a vacuum pump connected to a dry ice-cooled vacuum trap and two filter flasks containing NaOH pellets. The residue was dissolved in 200 μL 0.1 M HCl. An aliquot (10 μL) of each solution was analyzed by HPLC as described in Section 3.2.

Human NMs were isolated from SN and LC tissues and used in the experiments as described previously [6,7,10,28]. This study was approved by the Ethical Committee of the National Research Council of Italy-Institute of Biomedical Technologies (Segrate, Milan, Italy) and was carried out in agreement with the Policy of the National Research Council of Italy. SN-NM was isolated from a pool of five subjects with age range 73–85 years old. LC-NM was isolated from a pool of 35 subjects with age range 62–94 years old.

### 3.2. General Procedure for the Synthesis of 4-Amino-3-hydroxy and 3-Amino-4-hydroxyphenyl Derivatives ***8c**–**13c***

An appropriate phenol compound **8a**–**13a** (1 mmol) was mixed with 60% HNO_3_ (4.8 mmol). The resulting suspension was stirred at room temperature for 10 min for **8a** and **9a**, for 30 min for **10a**, **12a** and **13a**, or overnight for **11a**, leading eventually to an intensively dark red solution. HPLC analysis confirmed the absence of starting material and the presence of 3 major products corresponding most likely to nitro derivatives resulting from nitration at the *o*- and *p*-positions of the hydroxy group from the *m*-substituted phenols **8a**, **10a** and **12a**, but only one product from the *p*-substituted phenols **9a**, **11a** and **13a**. The crude nitrophenol **8b** or **9b** was diluted with water (20 mL) and applied onto a Dowex 50W-X2 (200–400 mesh) column (1.5 cm × 6 cm, H^+^ form, equilibrated with water). The column was washed with water (100 mL) and then the product was eluted with 2 M HCl (20 mL/fraction). Fractions containing the target compound were combined and evaporated to dryness to give a yellow powder. After the nitration of phenols **10a**, **12a** **and** **13a**, the reaction mixture (**10b**, **12b** and **13b**) was diluted with H_2_O (20 mL) and extracted with ethyl acetate (2 × 100 mL). The combined organic extract was washed with water (2 × 50 mL), dried with Na_2_SO_4_ and evaporated under reduced pressure. The nitration of **11a** afforded **11b** as an orange powder in the reaction mixture. After the filtration, the powder was washed with water and dried under reduced pressure to give a dark red powder **11b**.

The nitrophenol compounds **8b**–**13b** were then heated under reflux with 57% HI (10 mL) in the presence of 30% H_3_PO_2_ (1.0 mL). After 60 min, HPLC analysis confirmed the absence of the nitrated compounds and the appearance of products corresponding most likely to amino derivatives resulting from reduction to amine at the *o*- (and *p*-) positions of the hydroxy group. The mixture was evaporated to dryness, taken up in 0.1 M HCl (3 mL) and chromatographed on a Dowex 50W-X2 (200–400 mesh) column (1.8 cm × 31 cm, equilibrated and eluted with 2 M HCl). Fractions of 20 mL were collected and analyzed by UV spectrophotometry (200–400 nm) and by HPLC. The analytical conditions of HPLC are described in Section 3.2. After fractions of the target compound were collected, recrystallization of compounds **8c**–**13c** from ethanol (5 mL) plus ether (25 mL) and compound **14c** from 6 M HCl (3 mL) plus acetone (60 mL) gave colorless crystals as HCl salts.

### 3.3. HPLC Analyses of Aminophenols ***8c**–**13c***

The HPLC system consisted of a JASCO C_18_ column (JASCO Catecholpak; 4.6 mm × 150 mm; 7 µm particle size) and an EICOM ECD-300 electrochemical detector. The mobile phase used was 0.1 M sodium citrate buffer, pH 3.0, containing 1 mM sodium octanesulfonate and 0.1 mM Na_2_EDTA: methanol, 92: 8 (v/v) at 40 °C at a flow rate of 0.7 mL/min. The electrochemical detector was set at +500 mV *versus* the Ag/AgCl reference electrode. The retention times were 9.2, 9.7, 15.2, 16.2, 93.2 and 93.5 min for **10c**, **11c**, **8c**, **9c**, **12c** and **13c**, respectively. The retention times for **12c** and **13c** were shortened to 43.6 min using the above buffer: methanol, 80:20. It was impossible to separate **12c** and **13c** under the above HPLC conditions.

*4-Amino-3-hydroxyphenylethylamine (4-AHPEA)* (**8c**). Colorless crystals. Yield: 12%. Purity 99% by HPLC. ^1^H-NMR (DCl): δ = 2.85 (t, 2H, *J* = 8.0 Hz, CH_2_), 3.15 (t, 2H, *J* = 8.0 Hz, CH_2_), 6.80 (d, 1H, *J* = 4.0 Hz, CH), 6.86 (s, 1H, CH), 7.19 (d, 1H, *J* = 4.0 Hz, CH). ^13^C-NMR (D_2_O, *t*-butyl alcohol was added as an internal reference): δ = 28.34, 38.96, 115.45, 115.98, 119.60, 122.90, 137.74, 148.68. Elemental analysis: calculated for C_8_H_12_O_1_N_2_.2HCl. C, 42.68%; H, 6.27%; N, 12.44%; Cl, 31.50%; found: C, 43.25%; H, 6.07%; N, 12.43%; Cl, 32.76%. UV (0.1 M HCl) λ_max_ 274 nm (ε 2240) and 221 nm (ε 6,700).

*3-Amino-4-hydroxyphenylethylamine (3-AHPEA)* (**9c**). Colorless crystals. Yield: 84%. Purity 99% by HPLC. ^1^H-NMR (DCl): δ = 2.83 (t, 2H, *J* = 8.0 Hz, CH_2_), 3.13 (t, 2H, *J* = 8.0 Hz, CH_2_), 6.93 (d, 1H, *J* = 12.0 Hz, CH), 7.13 (s, 1H, CH), 7.15 (d, 1H, *J* = 12.0 Hz, CH). ^13^C-NMR (D_2_O, *t*-butyl alcohol was added as an internal reference): δ = 28.99, 39.87, 116.24, 117.60, 123.55, 128.25, 129.95, 148.29. Elemental analysis: calculated for C_8_H_12_O_1_N_2_.2HCl. C, 42.68%; H, 6.27%; N, 12.44%; Cl, 31.50%; found: C, 42.29%; H, 6.17%; N, 12.30%; Cl, 32.08%. UV (0.1 M HCl) λ_max_ 276 nm (ε 1,970) and 221 nm (ε 7,720).

*4-Amino-3-hydroxyphenylacetic Acid (4-AHPAA)* (**10c**). Colorless crystals. Yield: 12%. Purity 99% by HPLC. ^1^H-NMR (DCl): δ = 3.71 (s, 2H, CH_2_), 6.92 (d, 1H, *J* = 8.0 Hz, CH), 7.00 (s, 1H, CH), 7.33 (d, 1H, *J* = 8.0 Hz, CH). Elemental analysis: calculated for C_7_H_9_O_3_N_1_.HCl. C, 47.19%; H, 4.95%; N, 6.88%; Cl, 17.41%; found: C, 46.95%; H, 5.07%; N, 6.60%; Cl, 17.52%. UV (0.1 M HCl) λ_max_ 274 nm (ε 2,110) and 212 nm (ε 6,350).

*3-Amino-4-hydroxyphenylacetic Acid (3-AHPAA)* (**11c**). Colorless crystals. Yield: 52%. Purity 99% by HPLC. ^1^H-NMR (DCl): δ = 3.71 (s, 2H, CH_2_), 7.05 (d, 1H, *J* = 8.4 Hz, CH), 7.27 (d, 1H, *J* = 8.4 Hz), 7.28 (s, 1H). Elemental analysis: calculated for C_7_H_9_O_3_N_1_.HCl. C, 47.19%; H, 4.95%; N, 6.88%; Cl, 17.41%; found: C, 46.09%; H, 4.89%; N, 6.64%; Cl, 18.05%. UV (0.1 M HCl) λ_max_ 276 nm (ε 1,880) and 212 nm (ε 7,060).

*4-Amino-3-hydroxyethylbenzene (4-AHEB)* (**12c**). Colorless crystals. Yield: 4.7%. Purity 95% by ^1^H-NMR. ^1^H-NMR (DCl): δ = 1.18 (t, 3H, *J* = 7.6 Hz, CH_3_), 2.61 (dd, 2H, *J* = 7.6, 7.6 Hz, CH_2_), 6.91 (dd, 1H, *J* = 8.0, 1.6 Hz, CH), 6.95 (d, 1H, *J* = 1.6 Hz, CH), 7.27 (d, 1H, *J* = 8.0 Hz, CH). ^13^C-NMR (DCl): δ = 16.57, 29.78, 116.83, 117.56, 121.79, 125.61, 149.62, 151.51. Elemental analysis: calculated for C_8_H_11_O_1_N_1_.HCl.1/4H_2_O. C, 53.94%; H, 7.07%; N, 7.86%; Cl, 19.90%; found: C, 54.07%; H, 6.79%; N, 7.88%; Cl, 19.33%. UV (0.1 M HCl) λ_max_ 272 nm (ε 2,250) and 212 nm (ε 6,750).

*3-Amino-4-hydroxyethylbenzene (3-AHEB)* (**13c**). Colorless crystals. Yield: 42%. Purity 99% by HPLC. ^1^H-NMR (DCl): δ = 1.18 (t, 3H, *J* = 7.6 Hz, CH_3_), 2.60 (dd, 2H, *J* = 7.6, 7.6 Hz, CH_2_), 7.00 (d, 1H, *J* = 8.0 Hz, CH), 7.22 (s, 1H, CH), 7.24 (d, 1H, *J* = 8.0 Hz, CH). Elemental analysis: calculated for C_8_H_11_O_1_N_1_.HCl. C, 55.33%; H, 6.98%; N, 8.07%; Cl, 20.41%; found: C, 55.16%; H, 6.91%; N, 8.07%; Cl, 20.64%. UV (0.1 M HCl) λ_max_ 277 nm (ε 2,270) and 220 nm (ε 7,610).

*4-Amino-3-hydroxyphenylalanine (4-AHP)* (**14c**). Colorless crystals. Yield: 23%. Purity 99% by HPLC. ^1^H-NMR (DCl): δ = 3.11 (m, 2H, CH_2_), 4.07 (m, 1H, CH), 7.00 (d, 1H, *J* = 8.0 Hz, CH), 6.79 (d, 1H, *J* = 4.0 Hz, CH), 6.85 (s, 1H, CH), 7.22 (d, 1H, *J* = 4.0 Hz, CH). ^13^C-NMR (D_2_O, *t*-butyl alcohol was added as an internal reference): δ = 36.46, 55.33, 118.35, 118.60, 122.52, 125.70, 138.08, 151.37, 172.74. Elemental analysis: calculated for C_9_H_12_O_3_N_2_.2HCl.CH_3_COCH_3_. C, 44.05%; H, 6.16%; N, 8.56%; Cl, 21.67%; found: C, 44.77%; H, 5.98%; N, 8.49%; Cl, 20.47%. UV (0.1 M HCl) λ_max_ 215 nm (ε 6,840) and 275 nm (ε 2,370).

## 4. Conclusions

A general procedure for the synthesis of 4-amino-3-hydroxy- and 3-amino-4-hydroxyphenyl derivatives **8c**–**13c** was established based on the reduction of the nitro group to amine on the aromatic ring using 57% HI/H_3_PO_2_. This method is more convenient and generally applicable compared with other methods reported in the literature. Some degradation products of human NMs, SN-NM and LC-NM, were identified with these newly synthesized *o*-aminophenol compounds in HPLC determinations: the reductive HI hydrolysis of SN-NM and LC-NM afforded AHPEAs **8c**, **9c**, AHPAAs **10c**, **11c**, AHEBs **12c**, **13c** and AHPs **14c**, **15c** as degradation products. Then, by using this new degradative approach, we have found that SN-NM and LC-NM are derived not only from DA and NE, but also from several other catecholic precursors which are present in catecholaminergic neurons.
